# Histamine‐induced plasticity and gene expression in corticostriatal pathway under hyperammonemia

**DOI:** 10.1111/cns.13223

**Published:** 2019-09-30

**Authors:** Olga A. Sergeeva, Aisa N. Chepkova, Boris Görg, Filipe Rodrigues Almeida, Hans‐Jürgen Bidmon, Helmut L. Haas, Dieter Häussinger

**Affiliations:** ^1^ Molecular Neurophysiology, Medical Faculty Institute of Neural and Sensory Physiology Heinrich‐Heine University Duesseldorf Germany; ^2^ Medical Faculty Institute of Clinical Neurosciences and Medical Psychology Heinrich‐Heine University Duesseldorf Germany; ^3^ Clinic of Gastroenterology, Hepatology and Infectious Diseases Medical Faculty Heinrich‐Heine University Duesseldorf Germany; ^4^ Medical Faculty C.&O. Vogt Institute for Brain Research Heinrich‐Heine University Duesseldorf Germany

**Keywords:** histamine, hyperammonemia, striatum, synaptic plasticity

## Abstract

**Aims:**

Histamine H3 receptor (H3R) antagonists/inverse agonists increase vigilance. We studied brain histaminergic pathways under hyperammonemia and the transcriptome of receptors and their signaling cascades to provide a rationale for wake‐promoting therapies.

**Methods:**

We analyzed histamine‐induced long‐lasting depression of corticostriatal synaptic transmission (LLDhist). As the expression of dopamine 1 receptors (D1R) is upregulated in LGS‐KO striatum where D1R‐H3R dimers may exist, we investigated actions of H3R and D1R agonists and antagonists. We analyzed transcription of selected genes in cortex and dorsal striatum in a mouse model of inborn hyperammonemia (liver‐specific glutamine synthetase knockout: LGS‐KO) and compared it with human hepatic encephalopathy.

**Results:**

LGS‐KO mice showed significant reduction of the direct depression (DD) but not the long‐lasting depression (LLD) by histamine. Neither pharmacological activation nor inhibition of D1R significantly affected DDhist and LLDhist in WT striatum, while in LGS‐KO mice D1R activation suppressed LLDhist. Histaminergic signaling was found unchanged at the transcriptional level except for the H2R. A study of cAMP‐regulated genes indicated a significant reduction in the molecular signature of wakefulness in the diseased cortex.

**Conclusions:**

Our findings provide a rationale for the development of aminergic wake‐promoting therapeutics in hyperammonemic disorders.

## 1 INTRODUCTION

Histaminergic neurons are wake‐active pacemakers in the posterior hypothalamus which send their axons through the whole central nervous system (CNS).^1^ They carry autoinhibitory histamine receptors (H3R) whose blockade increases the level of histamine in the brain and consequently vigilance. An H3R antagonist/inverse agonist, pitolisant (WAKIX), has been introduced for the treatment of narcolepsy^2^ which is characterized by orexin‐deficiency. Histamine and orexins are responsible for the complementary and synergistic control of wakefulness.^3^ Further clinical studies are on their way testing the possible benefit of H3R antagonists in other neurodegenerative diseases characterized by fatigue and day‐time sleepiness. Although not accompanied by neurodegeneration, hyperammonemic conditions are associated with fatigue especially during extreme exercise or liver pathology.^4^, ^5^ Thus, patients suffering from hyperammonemia may benefit from symptomatic treatment with H3R antagonists/inverse agonists.

Histamine is an important regulator of a variety of brain functions including attention, motor activity, and learning.^1^ Histaminergic neurons are silent during sleep and the histamine synthesizing enzyme, histidine decarboxylase, shows maximal expression during active periods of the day.^6^ Data from patients with hepatic encephalopathy (HE) and from animal experiments demonstrate significant alterations in the striatal histamine content, expression of histamine receptors^7^, ^8^, ^9^ and in the modulation of corticostriatal neurotransmission by histamine.^10^ In the rodent striatum, histamine negatively modulates excitatory glutamatergic transmission from cortical and thalamic afferents acting through presynaptic H3 heteroreceptors.^11^, ^12^ Postsynaptic excitatory H2R mediates depolarization of striatal principle cells in the mouse by ca 7 mV.^12^ Through cAMP and PKA‐signaling, the H2R is known to trigger long‐term potentiation of synaptic transmission, neuronal excitability,^1^, ^13^ and oscillations^14^ in the hippocampus which impacts learning and memory. These actions contribute to the wake‐promoting function of histamine. Activation of presynaptic H3Rs not only negatively modulates glutamatergic inputs, but also reduces lateral inhibition between striatal projection neurons,^12^ thus improving signal/noise ratio during wakefulness. Moreover, H3Rs regulate striatal dopamine release^15^ and dopamine receptor‐mediated signaling.^16^ It is suggested that histamine‐dopamine interactions are important in several motor disorders, because in both Parkinson's disease and its experimental rat models the H3R expression and radioligand binding are altered in substantia nigra and caudate putamen.^17^, ^18^, ^19^

Some rare cases of Parkinsonism‐like disorders result from liver failure or liver dysfunction, thus classifying hepatic encephalopathy (HE) as a “basal ganglia disorder”.^20^ Some HE patients indeed profit from L‐Dopa medication.^20^ However, the hypodopaminergic phenotype of HE is not a general rule and a recent analysis of clinical studies did not recommend the use of dopaminergic agents in HE.^21^ Dopamine‐histamine interaction within the basal ganglia under hyperammonemia and in HE remains to be characterized. Our earlier studies showed a reduction of long‐lasting depression in response to histamine (LLDhist) in rats with portacaval anastomosis,^10^ but no change in LLDhist after the treatment of in vitro slices with ammonium chloride (5 mM) for 4‐8 hours.^22^ The symptoms of hyperammonemia alone do not reflect the symptoms of HE sufficiently well. Thus, in addition to hyperammonemia, elevated bile acids and glutamine levels in the blood of HE patients may play a role for the brain pathology.^23^, ^24^ In accordance, a more severe phenotype is achieved by combining hyperammonemic diet with bile duct ligation in rats.^25^ A recently developed mouse model displays hyperammonemia without changes in further blood components such as amino acids or inflammatory mediators.^26^ We reported that these mice with liver‐specific deletion of glutamine synthetase (“LGS‐KO”) show reduced exploratory activity in the open field and delayed habituation to a novel environment which goes along with alterations in glutamate‐receptor dependent synaptic plasticity in striatum and hippocampus.^27^ In addition, we found upregulated expression and function of the D1 dopamine receptor (D1R), but not the D2 dopamine receptor (D2R) in the striatum of LGS‐KO mice.^27^

As H3R can associate with D1R or/and D2R in the striatum building heterodimers with altered pharmacology,^28^, ^29^ we compare now histamine‐mediated plasticity in the corticostriatal pathway of WT and LGS‐KO mice and the modulatory action of D1R activation/inhibition. We analyze the transcriptome related to histaminergic neurotransmission in the striatum of WT and LGS‐KO mice. We discuss our results in comparison with previously published^30^, ^31^ cortical transcriptome data, obtained from patients with hepatic encephalopathy.

## 2 MATERIALS AND METHODS

### 2.1 Animals

Male liver‐specific GS knockout (LGS‐KO) mice and their wild‐type littermates (WT) at the age from 2 to 4 months were used. Gene‐targeted mice lacking functional hepatic GS were obtained and genotyped as described previously.^26^ In some experiments for the control of action of H3R‐ligands, we used histaminergic neurons from Tmt‐HDC mice.^32^ Slice preparation and cell‐attached voltage clamp recordings from histaminergic neurons are described in detail in De Luca et al.^33^ Mice were kept on a 12 hours day‐12 hours night light schedule with ad libitum access to food and water. All procedures were in compliance with the guidelines for the use of experimental animals, as given by the Directive 2010/63/EU of the European Parliament, the German “Tierschutzgesetz” (animal protection law) and approved by the local authorities (LANUV NRW: Landesamt für Umwelt, Natur und Verbraucherschutz Nordrhein Westfalen, Bezirksregierung Düsseldorf; permission number O58/91). All efforts were made to minimize the number of animals and their suffering.

### 2.2 Real‐time RT‐PCR

Striatal tissues were isolated from 1 to 3 horizontal brain slices, and total cellular mRNA was extracted using an mRNA isolation kit (Quickprep Micro mRNA Purification Kit, GE Health care, GB). Real‐time RT‐PCR was used to detect genotype‐related alterations in gene expression. Detailed description of the applied protocol has been presented previously.^27^ Primer sequences used for the amplification of histamine receptors (H1R and H3R) and HNMT (histamine N‐methyl transferase) were published previously.^32^, ^34^ Primers for the amplification of mouse H2R were as follows: up:5′‐GGCCAAGAAGTGAGTGTAGA‐3′and lo: 5′‐GAAGAGGTTGAGGATGGAAG‐3′ (as in^35^; expected PCR product size: 366 b.p.). Primers for the organic cation transporter 3 (OCT 3) were the same as in our previous study.^33^ Primers for mouse Homer1A,^36^ ARC, EGR1, and EGR2^37^ are listed in Table 1. Several house keeping genes (HKG): GAPDH, RPl13a, beta‐actin (actB), and Hsp90^27^ were compared in our initial experiments. Using selection criteria of Schmittgen and Livak (highest p value obtained from group comparison, unpaired *t* test), we selected RPl13a as HKG in experiments with mouse samples. Homer1A, ARC, EGR1, and EGR2 mRNA levels were quantified in total RNA preparations from *postmortem* human brain tissue from the European cohort by real‐time PCR as described recently.^38^ RNA quality was verified using the Agilent 2100 Bioanalyzer (Agilent Technologies), SYBR^®^ Green qPCR was performed on a ViiA7 real‐time PCR system (Applied Biosystems). Primers for the amplification were designed to match, when possible, the position of the mouse primers (see Table 1). Relative mRNA levels of a gene were estimated by the “2^−ΔΔCt^” method using human beta‐actin as a HKG.^38^ All Real‐time PCR reactions were validated by gel electrophoresis and by sequencing of selected products. The relative mRNA level encoding each gene in relation to HKG was estimated by the “2^−ΔΔCt^” method. Average 2^−ΔΔCt^ values for the WT genotype were taken as 1.0, and the individual values were expressed relative to this value. The fold change (FC) of mRNA according to the “2^−Ct^” method was calculated in each PCR run where cDNA of the same quantity (eg 100 ng in 1 µL) from at least 3 WT and 3 LGS‐KO mice was used as a template. Averages of two replicates per mouse were expressed as 2^−Ct^ and normalized on the average of all 2^−Ct^ values in the WT group.^39^

**Table 1:** Primers used for amplification of human (h) or mouse (m) transcripts

	Forward	Reverse	GeneBank ID
mHomer1A	GAAGTCGCAGGAGAAGATGGA	TGATTGCTGAATTGAATGTGTACC	NM_011982
hHomer1A	CTTCACAGGAATCCGCAGG	TTGGCTCTGAGTTCTGTGTCA	NM_017010059
mEGR1	ACCCTATGAGCACCTGACCAC	TATAGGTGATGGGAGGCAACC	NM_007913
hEGR1	GAGCACCTGACCGCAGAGTCTTT	CGGCCAGTATAGGTGATGGG	NM_001964
mEGR2	TGCTGATTCCTTTGATCGAG	AGGATGAGGCTCTGCTCACT	NM_010118
hEGR2	ACAATAGGTTGGGAGATGCTG	CTGTACAATGTCCCCCAAATC	NM_001321037
mARC	CAGCCCCCACAAGTTTATTT	TTGAGATCTCCAGGGTCTCC	NM_001276684
hARC	CCAGCCCCACAGATTTTATTTT	GTTGGCCACAGCCTCATGA	NM_015193
mActb	CGTGAAAAGATGACCCAGATCATGTT	GCTCGTTGCCAATAGTGATGACCTG	NM_007393
hActb	CGGGACCTGACTGACTACCTC	GAAGGAAGGCTGGAAGAGTGC	NM_001101
mHsp90	CTGCGAGTCGGACTTGGTCCG	TGCCTGAAAGGCAAAGGTCTCC	NM_008302
mGAPDH	TGTGTCCGTCGTGGATCTGA	TTGCTGTTGAACTCGCAGGAG	NM_001289726
mRPl13a	ATGACAAGAAAAAGCGGATG	CTTTTCTGCCTGTTTCCGTA	NM_009438

### 2.3 Agilent microarray analysis

Expression levels of selected genes were extracted from data sets acquired in two earlier studies by Agilent™ whole human genome microarray analysis.^30^, ^31^ We based our search on the gene list from the Synaptic plasticity RT^2^Profiler PCR array (Qiagen, Cat. no.PAMM‐126Z). Among 84 genes, we selected 18, which are expressed and characterized in the mouse and are related to cAMP/ PKA‐dependent signal cascades. We added to this list histamine and dopamine receptors and their signaling pathways. For microarray analysis, RNA was isolated from human *postmortem* brain tissue using a commercial kit (RNeasy mini kit, Qiagen) according to the instructions of the manufacturer. Agilent microarray analysis was performed by Miltenyi‐Biotech (Bergisch‐Gladbach, Germany) as described in.^30^, ^31^ Data sets were deposited at the public genomic data repository “Gene Expression Omnibus” (GEO, accession no. http://www.ncbi.nlm.nih.gov/geo/query/acc.cgi?acc=GSE41919 and http://www.ncbi.nlm.nih.gov/geo/query/acc.cgi?acc=GSE57193) from the National Center for Biotechnology Information (NCBI). The “European cohort” of patients with liver cirrhosis and HE consisted of *postmortem* human brain tissue taken from the intersection parietal to occipital cortex area. Tissue was provided by the body donor program of the Department of Anatomy of the University of Düsseldorf, Germany. For detailed information on the microarray analysis and the patient characteristics see.^30^ Statistical group analysis was performed using Student's *t* test with equal variances. In the “Australian cohort” *postmortem* brain tissue was taken from the fusiform gyrus from four control subjects and four patients with liver cirrhosis with HE.^31^ Tissue was provided by the Australian Brain Donor Programs NSW Tissue Resource Centre. Statistical analysis was performed using analysis of variance (ANOVA) and Tukey's multiple comparison post hoc test.

### 2.4 Electrophysiology

Horizontal slices (400 μm thick) were prepared with a vibratome as described previously.^27^ After at least two hours preincubation at room temperature, a single slice was transferred to a recording chamber perfused with artificial cerebrospinal fluid (aCSF) at a flow rate of 1.5‐2 mL/min at 32°C. Cortical fibers to the striatum were stimulated as previously described.^27^ After the initial testing of stimulus‐response relationships, the stimulus intensity was adjusted to induce a field response of approximately 60%‐70% of its maximal amplitude and the stimulation frequency was set to 0.033 Hz. Each experiment included a 15‐20 minutes period of control recording, application of chemical stimulus, and 60‐90 minutes of poststimulus period.

Signals were amplified, digitized at 10 kHz, stored on a hard disk of a PC using Clampex software of pClamp (Axon Instruments), and analyzed off‐line, using Clampfit and Excel software. Ten consecutive field responses (5‐minutes recordings) were averaged, the amplitude of corticostriatal postsynaptic peak response (N2 peak) was measured as in Chepkova et al^27^ normalized to baseline (the mean values for a 15‐20‐minutes control period) and plotted against time. The data were statistically analyzed using GraphPad Prism5 software and presented as mean ± SEM with *n* indicating the number of slices per group (each slice corresponded to one experiment, each group included slices from at least four animals). The Mann‐Whitney *U*‐test (MWT) was used (if not mentioned otherwise) to determine statistical significance between means as some data sets lacked normal distribution (Kolmogorov‐Smirnov test). Occurence of effect was compared with Fisher's exact probability test (FEPT) or chi‐square test. Differences were considered statistically significant when *P* < .05.

### 2.5 Drugs and chemicals

**Figure 1:**
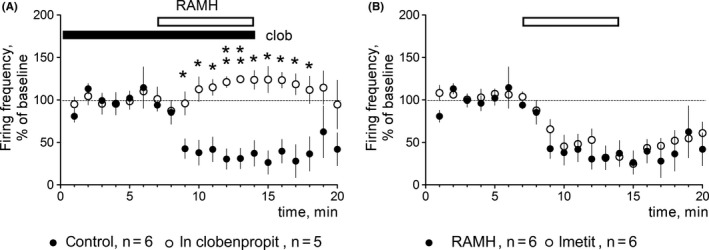
H3R pharmacology in histaminergic neurons of ventrolateral tuberomamillary nucleus of Tmt‐HDC mice, which express only H3R among 4 known histamine receptors. A, The H3R antagonist clobenpropit (clob) 20 µM abolishes inhibition of firing frequency of histaminergic neurons by 2 µM of RAMH (R‐(alpha)‐methylhistamine). Significant difference between data points is indicated by stars: *P < .05. **P < .01 (MWT). B, The H3R agonist imetit 3 µM inhibits firing frequency of histaminergic neurons to the same extent as RAMH 2 µM

The following substances were obtained from Tocris:

SCH23390 hydrochloride (Pubchem ID: 11957535), SKF38393 hydrobromide (Pubchem ID:12928470); clobenpropit dihydrobromide (Pubchem ID; 11213569), KT5720 (Pubchem ID: 9850141); imetit dihydrobromide (Pubchem ID: 11957573), Histamine dihydrochloride (Pubchem ID: 24277777) and R‐(alpha)‐methylhistamine dihydrochloride (Pubchem ID:24277778) were obtained from Sigma/RBI. Histamine was applied at 10 µM as previous studies demonstrated that this is an effective concentration to be studied in vitro.^11^, ^12^, ^14^, ^40^ To block H3R in slice recordings, previous studies used thioperamide 10 µM.^11^, ^12^ As this drug shows several additional activities,^41^ we decided to use another H3R antagonist, clobenpropit, which is more potent than thioperamide in vitro but less potent when applied in vivo due to the poor blood‐brain permeability.^42^ At 20 µM, clobenpropit was able to block maximal R‐(alpha)‐methylhistamine (RAMH, selective H3R agonist) responses in histaminergic neurons (see Figure 1A). Furthermore, we compared the action of the RAMH (2 µM) with the action of imetit (3 µM) and found no difference in the amplitude of response (Figure 1B). Concentrations of dopamine receptor modulators were chosen in accordance with previous studies in mouse.^43^, ^44^ All substances were diluted and stored as recommended by the provider. Working solutions were freshly prepared immediately before application.

## 3 RESULTS

**Figure 2:**
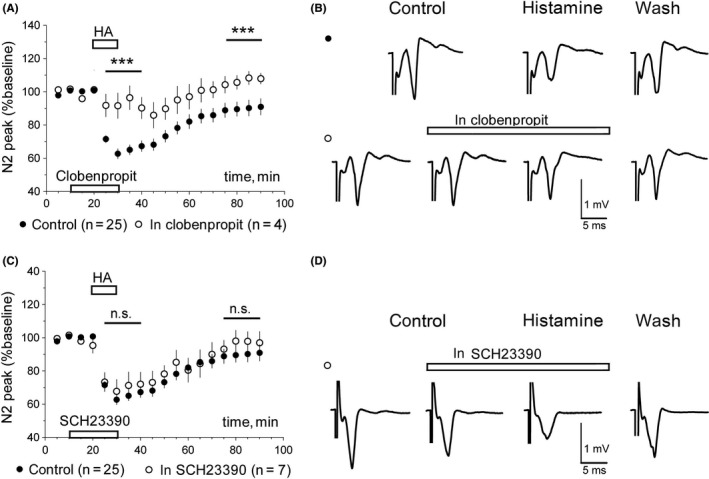
Pharmacological features of histamine‐induced long‐lasting depression (LLDhist) of corticostriatal synaptic transmission in wild‐type mice. A, LLDhist is abolished by the H3R antagonist clobenpropit (20 µM). Difference between data points at period indicated by a line was calculated with MWT, ***P < .005. B, Representative recordings of evoked corticostriatal field potentials in control experiments and in the presence of clobenpropit. Averages of responses collected during 10 min of (each) control period, 10 min of HA (histamine) application, and last 20 min of recording (washout) are shown. C, Averaged time course diagrams show no effect of the D1R antagonist SCH23390 (15 µM) on LLDhist. D, Averages of responses collected during 10 min of control period, 10 min of SCH23390 perfusion, 10 min of HA & SCH23390 application, and last 20 min of recording (washout) in one representative experiment

Application of 10 μM histamine to slice preparations from WT mice significantly inhibited corticostriatal neurotransmission in the vast majority of tested slices (n = 24 of 25) decreasing field response amplitude to 66.7 ± 1.4% (average of responses during 10 minutes histamine application followed by a 10 minutes‐washout period, 25th to 40th minutes of experiments) of baseline (20‐minutes period before application). This first phase of histamine‐induced response will be further referred to as “direct depression of corticostriatal transmission by histamine” or “DDhist”. In about half of the tested slices, this inhibition was sustained till 1 hour after histamine withdrawal with the average field response amplitude constituting 72 ± 2% of baseline for the last 20 minutes of recording (75th‐90th minutes of experiments). This phase is referred to as “long‐lasting depression of neurotransmission by histamine” or LLDhist. Low incidence of LLDhist in murine compared to rat striatum has been reported in a previous study.^11^ Both phases DDhist and LLDhist were significantly suppressed in the presence of the H3R antagonist clobenpropit at 20 μM, so that the average field responses amounted to 95.3 ± 7.6% and 109.5 ± 4.7% of baseline, respectively, compared to 66.7 ± 1.4% and 91.2 ± 2.2% in the control experiments (Figure 2). Occurrence of DDhist, but not of LLDhist, was significantly reduced under clobenpropit (*P* = .042, FEPT).

**Figure 3:**
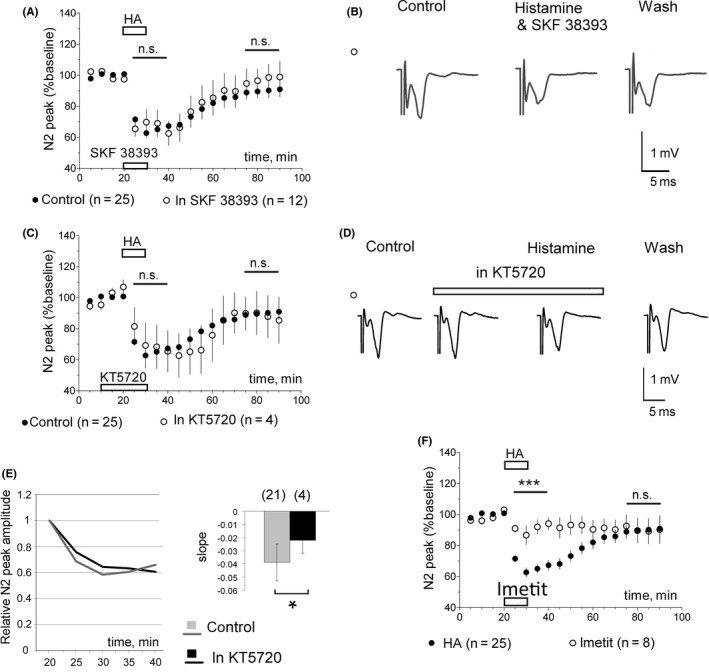
Pharmacological activation or inhibition of protein kinase A (PKA) does not affect LLDhist A, averaged time course diagrams show that the D1R agonist SKF 38393 (15 µM) has no significant impact on histamine‐induced depression of corticostriatal transmission. Difference between data points at time period indicated by a line was calculated with MWT, n.s.: not significant B, representative recordings of evoked corticostriatal field potentials in control (10 min‐), during coapplication of histamine and SKF 38393 (10 min‐) and last washout period (20 min‐averages). C, The PKA inhibitor KT5720 (1 µM) does not affect LLDhist. D, Averages of responses collected during 10 min of control period, 10min of KT5720 perfusion, 10 min of histamine & KT5720 application and the last 20min of recording (washout) in one representative experiment. E, Analysis of the onset kinetics of histamine (HA) 10 µM response in control and in the presence of KT5720 shows significantly slower development of DDhist in presence of the PKA antagonist (left: averages of two groups, right: bar histograms with number of slices analyzed above it. *P* < .05, MWT). F, Imetit 3 µM, applied to the corticostriatal slices, is significantly less effective (***P < .005, MWT) than histamine (HA, 10 µM) in the induction of direct depression of corticostriatal transmission and does not differ from histamine in the induction of long‐lasting depression

As dimerization between H3R and D1R in the striatum was suggested by Ferrada et al^29^ and we found previously that the D1R expression is upregulated in the LGS‐KO striatum,^27^ we tested whether the D1R agonist SKF 38393 or the D1R antagonist SCH23390 modulate histamine effects. No significant difference was observed in either DDhist or LLDhist when histamine was applied together with one of these compounds in WT slices (Figures 2 and 3). Histamine‐induced long‐lasting depression (LLDhist) was not significantly affected by inhibition of the cAMP‐dependent protein kinase (PKA) whose activity is known to be regulated through D1R (Figure 3C,D). We noticed that PKA inhibition by KT5720 (1 µM) delayed the DDhist maximum. To analyze this effect, we calculated the slope of onset with a linear regression. All data points were scaled to the control and only experiments with inhibition larger than 20% were considered. Slopes of control responses and responses in the presence of KT5720 differed significantly (Figure 3E). As direct depolarization of striatal medium spiny neurons by histamine through H2R was previously reported^12^ and our experiments with KT5720 indicated a H2R (PKA)‐dependent component, we applied the selective H3R ligand imetit (3 µM), which showed the same action as RAMH on histaminergic neurons (Figure 1). Imetit‐induced direct depression of corticostriatal transmission was significantly smaller compared to the response to histamine 10 µM (Figure 3F) whereas long‐lasting depression did not differ. Thus, LLDhist is solely dependent on H3R, whereas DDhist involves H2R and H3R activation.

**Figure 4:**
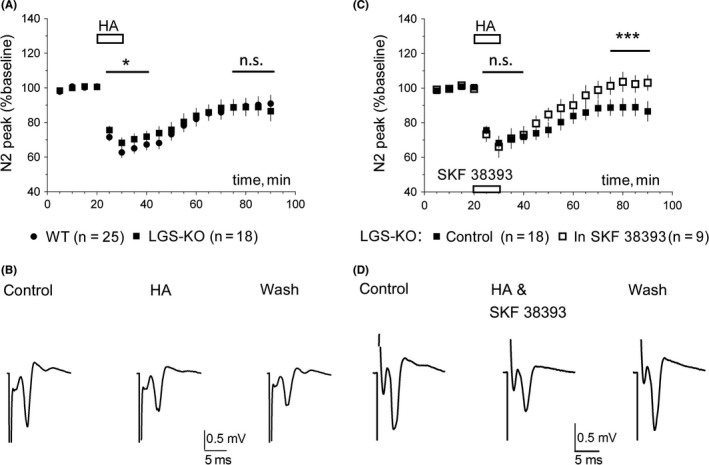
Histamine‐induced depression (LLDhist) of corticostriatal synaptic transmission in LGS‐KO striatum. A, Initial inhibition of corticostriatal transmission by histamine is significantly smaller in LGS‐KO mice compared to the WT. Sustained inhibition (minutes 75‐90 of recordings) does not differ between genotypes. Difference between data points at time period indicated by a line was calculated with MWT, *P < .05; n.s.: not significant. B, Representative recordings of field potentials and their LLDhist in LGS‐KO mouse. Averages of responses collected during 10 min of control period, 10 min of histamine application, and last 20 min of recording (washout). C, Averaged time course diagrams show that combined application of histamine (10 µM) with the D1R agonist SKF 38393 (10‐15 µM) significantly accelerates recovery from inhibition in LGS‐KO mice (***P < .005, MWT). D, Representative averages of field responses in control (10 min), during histamine & SKF 38393 coapplication (10 min), and 20 last minutes of recording (washout) in LGS‐KO mouse

**Figure 5:**
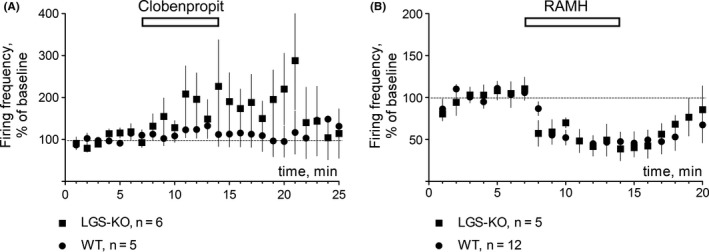
Modulation of LGS‐KO and WT histaminergic neurons by H3R ligands. Histaminergic neurons of the ventrolateral tuberomammillary nucleus, identified through excitation by the H3R antagonist clobenpropit 20 µM A, and/or inhibition by the H3R agonist RAMH 2 µM B, are similarly modulated in mice. No significant difference between data points (MWT)

LGS‐KO mice displayed a significantly less pronounced DDhist than that found in the WT striatum (Figure 4A,B). The mean amplitude of DDhist in slices from LGS‐KO mice constituted 71 ± 1% of baseline vs 66 ± 1% in slices from WT mice (*P* = .03, MWT). LGS‐KO striatal slices did not differ from WT either in the occurrence (n = 11 of 23 WT and n = 8 of 18 LGS‐KO slices, *P* = .74 chi‐square test) or the magnitude of LLDhist (to 72 ± 2% and 68 ± 2% of baseline in WT and KO, respectively, *P* = .6, MWT). In LGS‐KO mice, activation of D1R with the potent and selective agonist SKF38393 did not affect DDhist but significantly suppressed the maintenance of LLDhist (Figure 4C,D). We did not detect differences between LGS‐KO and WT mice either in inhibition of firing of histaminergic neurons by RAMH or in effects of clobenpropit 20 µM (Figure 5).

**Figure 6:**
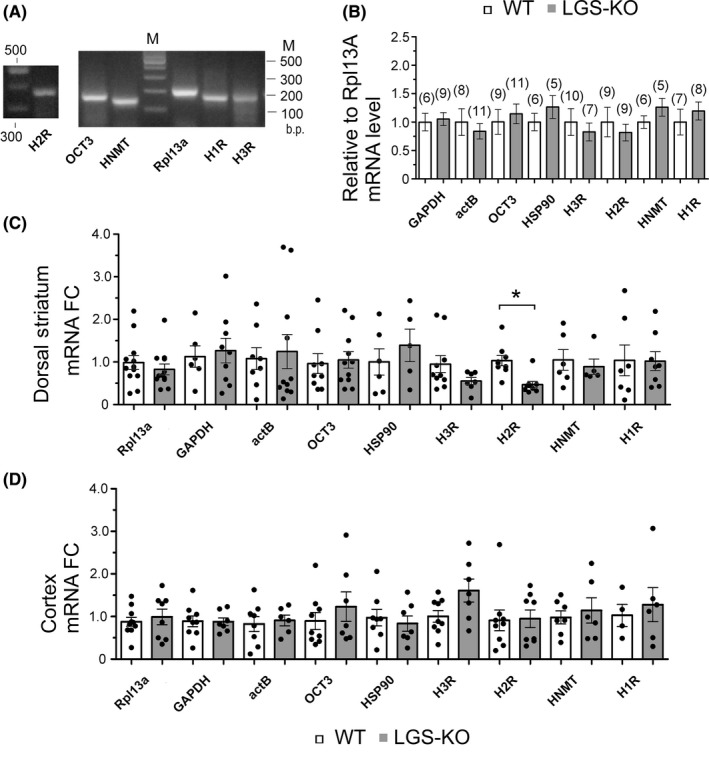
Transcription of histaminergic markers in mouse brain. A, Representative PCR products of expected size visualized after real‐time PCR on gel‐red stained agarose gel. M: DNA size marker (100 b.p. ladder). B, Bar histograms represent average and SEM from all real‐time PCR experiments. All reactions were normalized on the expressional level of Rpl13a followed by calibration on the WT mouse striatum (2^−∆∆Ct^ method). Numbers of animals are given above the bars. C, Fold change (FC) datapoints (2^−Ct^ method), one per mouse, each is an average of 3‐12 experiments. Medians of 18 groups do not differ significantly (*P* = .428, one‐way ANOVA with Kruskal‐Wallis test). Dunn's multiple comparison test revealed difference between H2R expression in dorsal striatum of LGS‐KO vs WT (*P < .05), whereas expression of the house keeping genes Rpl13a, Hsp90, GAPDH and actB did not differ. D, Fold change (FC) datapoints (2^−Ct^ method) from mouse cortex. Same tests as in (C) reveal no difference between genotypes

Transcription of several genes related to the histaminergic pathway was compared between WT and LGS‐KO striatum. Using the “2^−ΔΔCt^” method, we found no significant difference in mRNA levels for the histamine receptors H1, H2 and H3 (HRH1, HRH2, HRH3 nomenclature is used for the human and H1R, H2R, H3R for the mouse brain), histamine N‐methyl transferase (HNMT), and organic cation transporter 3 (OCT3)^45^ (Figure 6A,B). Analyzing only experiments with the same input quantity with the “2^−Ct^” method, we found decreased H2R‐mRNA level in LGS‐KO striatum (*P* < .05, one‐way ANOVA with Dunn's multiple comparison test, Figure 6C). The same analysis performed with mouse cortical samples did not reveal any difference in transcripts encoding for histamine receptors (Figure 6D).

**Figure 7:**
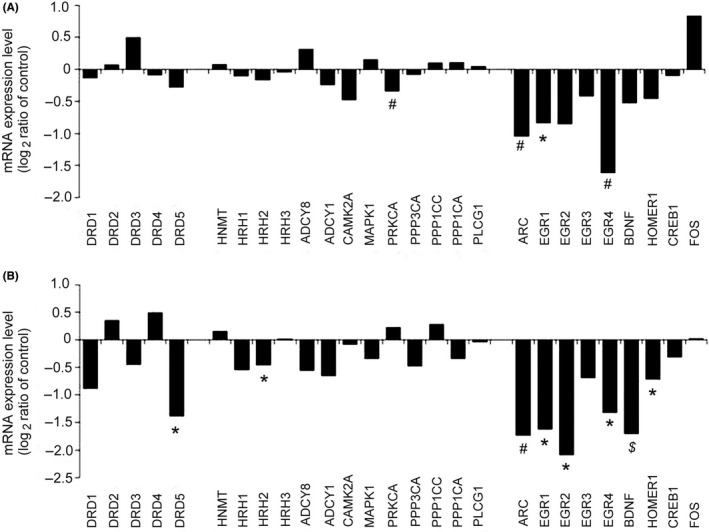
Expression changes of genes related to dopamine and histamine signaling in *postmortem* brain tissue from the cerebral cortex of patients with liver cirrhosis and HE. Gene expression changes were measured by microarray analysis in two independent patient cohorts: one from Europe A, gene expression was analyzed with Student *t* test^30^; and another from Australia B, gene expression was analyzed with one‐way ANOVA due to the study design.^31^ In A and B, gene expression changes are given relative to the respective controls. *P < .05; #*P* ≤ .01; $*P* ≤ .001

**Figure 8:**
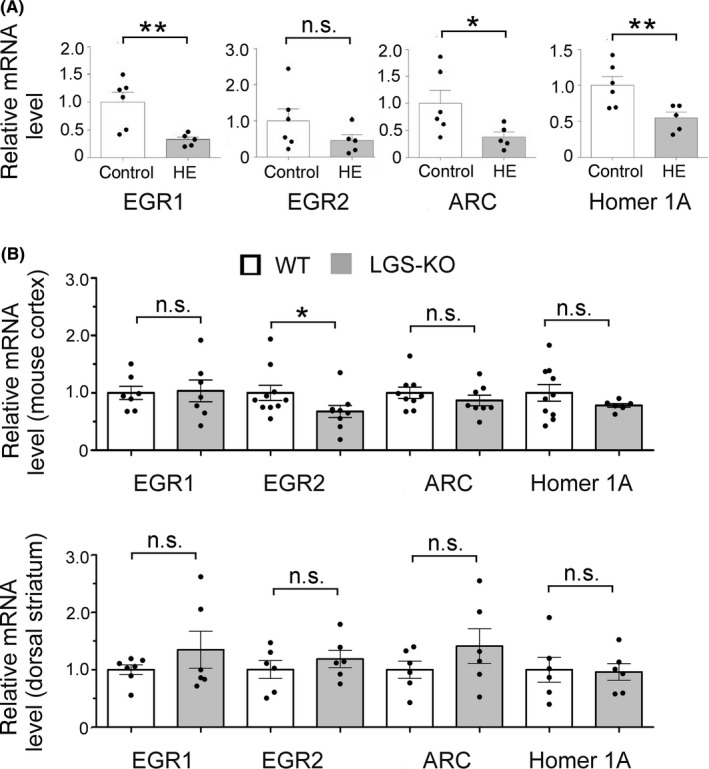
Real‐time PCR analysis of “molecular signature of wakefulness” in human cortex *postmortem* (A) and mouse (B) cortex and striatum of WT and LGS‐KO mice. A, Same samples as for microarray experiments shown in Figure 5A were amplified and analyzed with the “2^−ΔΔCt^” method, using beta‐actin expression as a reference. Note that results similar to those from gene array were obtained. *P = .05, **P = .01 (As values in each group fulfilled criteria of normal distribution (Kolmogorov‐Smirnov normality test), the unpaired *t* test was applied for the group comparison). B, Transcriptional changes in LGS‐KO mouse cortex (upper plot) or dorsal striatum (lower plot) in comparison to WT (significant difference is indicated as *P < .05 (MWT)

In search for further gene transcripts which might be affected by hyperammonemia and involved in histamine‐ or dopamine‐mediated synaptic plasticity, we screened for relevant genes in the cortical transcriptome of HE patients *postmortem*. We found similarities between Australian and European cohorts in the reduced expression of ARC (activity‐regulated cytoskeleton‐association protein), EGR1 (early growth response protein 1), and EGR4 but did not detect changes in HNMT (histamine N‐methyltransferase: a correlate of histaminergic activity^46^). Transcript levels of HRH2, HOMER1, BDNF (brain derived neurotrophic factor), EGR2, and DRD5 (dopamine receptor D5) were significantly reduced in Australian but not in European HE patients (Figure 7A,B). Our real‐time PCR validation of whole transcriptome data^30^ showed differences similar to those obtained with microarray analysis (Figure 8A), except for Homer1A, whose down‐regulation detected with our primers (Table 1) was significant (Figure 8A). All reactions were normalized on the beta‐actin (actB) level as this HKG showed stable expression with small variance in our samples (Ct = 23.1 ± 0.3 (n = 6) in control vs 22.8 ± 0.7 (n = 5) in HE, *P* = .7, Student's *t* test).

We quantified expression of ARC, EGR2, and Homer1A in cortical and striatal samples of LGS‐KO and WT mice (Figure 8B). The fold change (FC, “2^−Ct^” method) analysis did not reveal any difference between the two genotypes. When reactions were normalized on Rpl13a (“2^−ΔΔCt^” method), we detected a significantly reduced expression of EGR2 in LGS‐KO mouse cortex (*P* = .02, MWT).

## 4 DISCUSSION

This study shows that mice with inborn hyperammonemia (LGS‐KO) display lower sensitivity of corticostriatal neurotransmission to histamine (significantly lower DDhist) without any significant changes of histamine‐induced corticostriatal plasticity (LLDhist) and in the striatal expression of genes relevant for synaptic plasticity, except for a slight reduction in H2R expression, which may impact DDhist. Histamine‐induced effects in WT striatum were abolished by H3R antagonism in agreement with previous studies^11^, ^12^ but turned out to be insensitive to either activation or inactivation of D1 dopamine receptors with pharmacological tools. In contrast, activation of D1R significantly suppressed LLDhist in LGS‐KO striatum which might be associated with upregulation of D1R in LGS‐KO.^27^ Although corticostriatal LLDhist was found to be independent of the D1R‐cAMP‐protein kinase A (PKA) pathway, DDhist had a tendency to be delayed, indicating involvement of postsynaptic H2R. Moreover, investigating expression of cAMP‐regulated genes in the diseased cortex of hepatic encephalopathy patients, we found a significant reduction in the molecular signature of wakefulness^47^ in European and Australian cohorts of patients *postmortem* and the histamine H2 receptor (HRH2) in the Australian cohort (with one‐way ANOVA).

Ferrada et al^29^ have shown dimer formation between H3R and D1R in mammalian transfected cells with Bioluminescence Resonance Energy Transfer and binding assays. In the D1‐H3 dimer, signaling via each receptor was blocked not only by a selective antagonist but also by an antagonist of the partner receptor. Moreover, the H3R alone does not couple to MAPK (mitogen‐activated protein kinase) signaling, whereas, after dimerization with D1R, its stimulation leads to extracellular signal‐related kinase (ERK)1/2 phosphorylation in the striatum, which is absent in D1R knockout mice.^29^, ^48^ ERK1/2 phosphorylation by RAMH is blocked during coapplication with a D1R antagonist (SCH23390).^29^ Direct electrophysiological evidence is missing so far for dimerized H3R pharmacology in mammalian striatum where both receptor types are highly expressed.^12^ We did not detect a significant influence of D1R antagonism on H3R‐mediated LLDhist in WT mice. This may be explained by the involvement of other signaling cascades such as inhibition of cAMP formation by H3R^28^ or by the absence of D1‐H3 dimerization at the presynaptic sites of cortical afferents, where H3R induced LLDhist occurs.^12^ We found no difference in LLDhist recorded in normal aCSF and in the presence of the protein kinase A inhibitor KT5720. Thus, it is likely that presynaptic H3Rs directly suppress Ca2+ inflow through voltage‐gated Ca2+ channels.^40^ Do excitatory D1Rs exist at the same presynaptic sites? A recent study in cortical cultures showed that glutamate release can be stimulated through D1Rs, an effect abolished in the presence of the protein kinase A inhibitor KT5720 (200nM).^49^ We took advantage of the slightly upregulated D1R expression in LGS‐KO mice^27^ and studied the influence of a D1R agonist on H3R‐mediated plasticity: a D1R agonist, which stimulates cAMP production^29^, ^48^ counteracted LLDhist in LGS‐KO but not in WT mice indicating stronger signaling through D1R in mice with inborn hyperammonemia. Additivity of D1R‐ and H3R‐mediated effects suggests spatial separation of the receptors. Thus, our data do not support the existence of H3R‐D1R dimers; at least such receptors do not modulate corticostriatal glutamatergic transmission at the presynaptic site. Besides corticostriatal and thalamostriatal glutamatergic synapses H3Rs also modulate striatal feedback inhibition through inhibitory connections between the principle medium spiny neurons (MSN),^12^ abolishing this inhibition during wakefulness. Thus LLDhist together with facilitation of glutamatergic thalamic input and interruption of lateral inhibition between MSN through H3R may allow gain control of sensory information and prevent input saturation during wakefulness,^12^ when histaminergic neurons are active.^1^ Further studies should clarify the role of H3R‐D1R dimerization in different electrophysiologically defined striatal synapses.

Transcriptome analysis revealed down‐regulation of cAMP‐PKA‐dependent immediate early genes, which are involved in synaptic plasticity, growth and rearrangement/rescaling of synapses,^47^, ^50^, ^51^ in the cortex of HE patients. This reduction must coincide with the decreased vigilance, attention, and cognitive abilities of affected individuals, especially as some of them died in hepatic coma.^30^ We found in a mouse model of inborn hyperammonemia, showing only mild pathology, a change in EGR2 expression similar to that seen in human HE. Thus, transcription of this gene might be affected by hyperammonemia or other factors associated with this disease and could be used as an early marker. Upregulation of mRNA encoding for the early growth response proteins (EGR1, EGR2) was previously reported in mouse cortex in response to stress^52^ or weaning^53^ and down‐regulation in response to metamphetamine abuse.^37^ Lozeva et al^9^ reported increased histamine levels in *postmortem* cortex of HE patients and increased occupancy of H3R. We did not detect in mouse or man brain tissue samples changes in the expression of the histamine degrading enzyme HNMT, whose level correlates with the histaminergic activity in the brain.^46^ We found no changes in histamine receptor expression except for the down‐regulation of cAMP‐stimulating H2R in Australian HE patients and in the mouse striatum (with “2^−Ct^” method). Although we obtained only cortical samples from HE patients, mouse brain samples showed region‐specific gene alterations under hyperammonemia, which may play a role for the neurological symptoms. Taking into consideration subtle changes in histamine‐plasticity and gene expression in the histaminergic system of hyperammonemic mice and in the end‐stage of human HE as well as unimpaired H3R signaling, H3R antagonists/inverse agonists can be suggested as possible vigilance‐increasing therapeutics.

Wake‐promoting medication with H3R antagonism is indicated to combat day‐time sleepiness. We show that in inborn hyperammonemia accompanied by fatigue and motor disturbances, transcription of H3R, and histamine‐induced long‐lasting depression (LLDhist) are not changed. We demonstrate a misbalance between modulatory functions of histamine (decreases) and dopamine (increases through D1R) in mice with inborn hyperammonemia. These findings provide a rationale for the development of therapies directed toward aminergic neurotransmission.

## CONFLICT OF INTEREST

The authors declare that they have no conflict of interest. All applicable international, national, and institutional guidelines for the care and use of animals were followed.
